# Awareness and reported violations of the WHO International Code and Pakistan's national breastfeeding legislation; a descriptive cross-sectional survey

**DOI:** 10.1186/1746-4358-3-24

**Published:** 2008-10-17

**Authors:** Mihretab Salasibew, Ayyaz Kiani, Brian Faragher, Paul Garner

**Affiliations:** 1 Liverpool School of Tropical Medicine, Liverpool, UK; 2 TheNetwork for Consumer Protection, Islamabad, Pakistan

## Abstract

**Background:**

National legislation in Pakistan adopted the International Code of Marketing of Breastmilk Substitutes in 2002 to restrict the promotion of infant formula feeding. Our objectives were to assess health professionals' awareness of this law in urban government hospitals and describe their reports of violations, including receiving free samples, gifts and sponsorship.

**Methods:**

Structured interviews were conducted with health staff between July and August 2006 at 12 urban government hospitals in Islamabad, Rawalpindi and Peshawar including paediatricians, obstetricians, nurses, resident doctors, midwives and lady health visitors (LHVs).

**Results:**

Of the 427 health workers interviewed, the majority were not aware of the national breastfeeding law (70.5%; n = 301) or the International Code (79.6%; n = 340). Paediatricians, and staff who had been working for 10 years or more, were more likely to be aware of the law [OR = 7.00, 95% CI 3.12, 15.7 (paediatricians); OR = 2.48, 95% CI 1.45, 4.24 (10 years working)].

More than one third (38.4%, n = 164) had received small gifts such as pens, pencils and calendars; 12.4% (n = 53) had received sponsorship for training or conferences; and 15.9% (n = 68) had received free samples of infant formula from the Companies. Staff who were aware of the law were also more likely to report receiving gifts (OR = 1.64, 95% CI 1.08, 2.51) and free samples (OR = 1.86, 95% CI 1.09, 3.19).

**Conclusion:**

Most hospital health professionals were unaware of national breastfeeding legislation in Pakistan, and infant formula companies were continuing to flout the ban on gifts, free samples and sponsorship for health staff.

## Background

Artificial feeding in infants is associated with a higher risk of gastrointestinal and lower respiratory tract infections [1]. Further evidence shows long term risks to obesity, type-II diabetes, higher blood pressure and higher total cholesterol level [2]. In developing countries, the risks of poor health from bottle feeding are particularly marked. Global recommendations are for exclusive breastfeeding for the first six months of child's life. Thereafter, continued breastfeeding with the addition of nutritionally adequate complementary foods is recommended until at least a child's second birthday [3]. Despite these guidelines, the infant food industry continues its unethical marketing practices [4].

WHO recommended the International Code of Marketing of Breastmilk Substitutes during the 34^th ^World Health Assembly in Geneva in 1981 to control such marketing practices [5]. So far, 72% of the 193 recently surveyed countries have taken some kind of action to implement the International Code [6]. In South Asia, countries adopted the Code as SAARC (South Asian Association for Regional Cooperation) model code on Breastfeeding and Young Child Nutrition in their 3^rd ^Ministerial conference held in Rawalpindi, Pakistan in August 1996 [7].

Pakistan is among the 118 countries that voted in favour of adopting the International Code in May 1981. A National Code monitoring survey was conducted in 1998 in 33 cities and towns in Pakistan. It was found that not a single company marketing infant food or feeding products was abiding by the International Code in its entirety [8]. As a result, the country enacted a national breastfeeding law in October 2002 to enforce implementation of the Code [9].

This legislation adopts all provisions of the International Code and subsequent WHO resolutions. It prohibits promotion of any milk produced as partial or total replacement for mother's milk or represented as a complement to mother's milk to meet the growing nutritional needs of an infant. Promotions of feeding bottles, teats, valves for feeding bottles, pacifiers or nipple shields are also forbidden. Health workers are not permitted to receive any offers including gifts, free samples and sponsorship from these companies [9].

The National Infant Feeding Board monitors implementation and companies or individuals breaching the law may face prosecution and suspension of licence. Further sanctions include a maximum of two years imprisonment or between fifty to five hundred thousand rupees (€449 to €4,496 as of 01 September 2008) fine or both [9]. However, the representation of companies as members of the board remains controversial and of major concern for many breastfeeding campaigners in Pakistan [10].

So far, little work has been done in evaluating the degree of success of the national breastfeeding legislation. This small study aimed to evaluate the implementation of the national legislation [9] entitled, Protection of Breastfeeding and Young Child Nutrition Ordinance 2002, in urban government hospitals in Pakistan.

## Methods

Our objectives were to assess awareness among health workers about the existence of the law and to describe violations of the law in urban government hospitals. The main outcome measures were the proportion of health workers who reported they were aware of the law or the Code, and the proportion of health workers who claimed they received gifts, free samples or sponsorship from the infant formula companies.

We purposefully selected 12 urban government hospitals in three major cities in Pakistan for convenience reasons: 3 hospitals in Islamabad, 6 hospitals in Peshawar and 3 hospitals in Rawalpindi. Thus, we conducted the study in the capital city Islamabad and in 2 of the 4 Provinces of Pakistan which were Rawalpindi from Punjab province and Peshawar from North West Frontier Province.

Professional health staff who were working in paediatric inpatient wards, paediatric outpatient wards, the delivery suite, antenatal and postnatal care wards on the day that the interviews were conducted were included in the study. Specialist medical consultants, such as radiologists, who were neither paediatricians nor obstetricians, were excluded from the study as they might not be involved in routine breastfeeding consultations. Administrative staff, laboratory staff and x-ray technicians were also excluded.

Health workers were allocated to each group based on their role: nurses, midwives/Lady Health Visitors (LHVs), paediatricians, obstetricians and resident doctors (either in paediatrics or obstetrics). Data were collected between July and August 2006 through interviews using a structured questionnaire. MS, with two local research assistants, interviewed all health workers fulfilling the inclusion criteria and administered the questionnaires simultaneously.

We asked health workers how the companies were promoting formula in their hospitals, if at all, and whether they themselves had ever received any gifts, free samples or sponsorship from the companies. We verified responses by asking some of the health workers to show us the gifts and free samples they received from the companies. Towards the end of the questionnaire we asked health workers whether they were aware about the existence of the law or the Code but we did not assess their in-depth knowledge about its contents (See additional file 1: Questionnaire).

Statistical analyses were conducted using the SPSS version 15 and Epi-Info version 3.3.2 statistical software packages. Comparisons between pairs of variables were made using the Fisher's exact test; results were summarised as odds ratios with their 95% confidence intervals. Multiple logistic regression models were used to examine the combined effect of awareness about the law, occupation, city of residence, length of experience and level of patient workload on the likelihood that a respondent had received either gifts or free formula samples.

The study protocol was granted approval from the Research Ethics Committee in Liverpool School of Tropical Medicine, UK and permission was also obtained locally from each hospital included in the study. All interviewed health workers gave their consent to be interviewed anonymously while the names of their hospitals remained confidential.

## Results

We interviewed 427 health workers from 12 urban government hospitals between July and August 2006; this constituted almost all (98.3%) of the eligible health workers in these hospitals. The mean age of the respondents was 29.7 (Standard deviation = 8.2) years and the majority (78.2%, n = 334) were female. A total of 149 (34.9%) were currently working in the paediatric inpatient ward, 124 (29.0%) were involved in the delivery suite and postnatal care, 99 (23.2%) were working in the antenatal ward, and 55 (12.9%) were working in the paediatric outpatient ward. The median number of patients attended by respondents was 25 (range: 4 – 200) and most (84.5%, n = 361) had less than 10 years work experience (median = 2 years, range: 1 – 32).

Overall, 131 (30.7%) of the respondents were nurses, 114 (26.7%) were resident doctors, 70 (16.4%) were paediatricians, 60 (14.1%) were midwives or LHVs, and 52 (12.2%) were obstetricians (Table 1). However, these proportions differed significantly between the three cities covered by this study (Fisher's exact test, p < 0.01).

**Table 1:** Distribution of respondents by city of residence

	**City**			
		
**Occupation**	**Islamabad** **N (%)**	**Peshawar** **N (%)**	**Rawalpindi** **N (%)**	**Total** **N (%)**
Obstetricians	17 (12.1)	14 (8.2)	21 (17.9)	52 (12.2)
Midwives/LHVs^1^	0 (0)	59 (34.7)	1 (0.9)	60 (14.1)
Nurses	55 (39.3)	39 (22.9)	37 (31.6)	131 (30.7)
Paediatricians	28 (20.0)	27 (15.9)	15 (12.8)	70 (16.4)
Resident doctors	40 (28.6)	31 (18.2)	43 (36.8)	114 (26.7)

Total	140	170	117	427

^1^LHV: lady health visitor

### Awareness about the law/Code

Most respondents (70.5%, n = 301) were unaware of the existence of the national breastfeeding law of Pakistan. Paediatricians were significantly more likely, and midwives significantly less likely, than other groups of health workers to be aware of this law.

Respondents in Islamabad were significantly more likely than those in Peshawar to be aware of the law (OR = 2.42, 95% CI 1.42, 4.14). Experienced health workers were significantly more likely to be aware of the law than their less experienced colleagues (Table 2).

**Table 2:** Characteristics of health workers who were aware about law

**Variable**	**Aware/total (%)**		**Odds ratio (95% C.I.)**	
			
			**Unadjusted**^**1**^	**Adjusted**^**2**^
All	126/427	(29.5)		

**Occupation**				
Obstetricians	13/52	(25.0)	1.00	1.00
Midwives/LHVs^3^	2/60	(3.3)	0.10 (0.02, 0.48)	0.16 (0.33, 0.81)
Nurses	35/131	(26.7)	1.09 (0.52, 2.29)	0.98 (0.46, 2.10)
Paediatricians	49/70	(70.0)	7.00 (3.12, 15.7)	7.13 (3.11, 16.4)
Resident doctors	27/114	(23.7)	0.93 (0.44, 2.00)	1.05 (0.48, 2.28)

**City**				
Islamabad	54/140	(38.6)	1.00	1.00
Peshawar	35/170	(20.6)	0.41 (0.25, 0.68)	0.54 (0.30, 0.99)
Rawalpindi	37/117	(31.6)	0.74 (0.44, 1.24)	0.82 (0.47, 1.43)

**Experience**				
<10 years	95/361	(26.3)	1.00	1.00
≥ 10 years	31/66	(47.0)	2.48 (1.45, 4.24)	1.99 (1.05, 3.76)

**Patients**				
<50 per day	94/341	(27.6)	1.00	1.00
≥ 50 per day	32/86	(37.2)	1.56 (0.95, 2.56)	1.01 (0.57, 1.79)

^1^Univariate analysis, each factor considered singly

^2^Multiple logistic regression analysis, with all factors considered simultaneously

^3^LHV: lady health visitor

The majority of health workers 340 (79.6%) were also not aware of the existence of the International Code. Those health workers who were aware of Pakistani law were more likely to be aware of the International Code as well (OR = 23.0, 95% CI 12.4, 42.5).

### Violations

More than one-third of the 427 health workers interviewed (38.4%, n = 164), confirmed that they had received gifts such as pens, pencils and calendars labelled with the names of infant formula companies. In addition, 68 (15.4%) had received free samples of infant formula, and 53 (12.4%) confirmed that they had received sponsorship for attending conferences or training sessions.

Highly qualified health workers (obstetricians, paediatricians and resident doctors) were significantly more likely to have received gifts than nurses and midwives (OR = 9.52, 95% CI 5.66, 16.10). Respondents who were aware of the Pakistani law were significantly more likely than their colleagues who were unaware, to have received gifts. Of the 126 health workers who were aware of the law, almost half (46.8%, n = 59) confirmed that they had received gifts. However, this difference became statistically non-significant when adjusted for the effects of occupation, city of residence, experience and patient workload.

Considered alone, no differences were found between cities, but when adjusted for other factors, respondents in Peshawar emerged as being significantly more likely to have received gifts than those from the other two cities. Receipt of gifts was not significantly associated with either years of experience or level of patient workload (Table 3).

**Table 3:** Characteristics of health workers who received gifts

**Variable**	**Received/total (%)**		**Odds ratio (95% CI)**	
			
			**Unadjusted**^**1**^	**Adjusted**^**2**^
All	164/427	(38.4)		

**Occupation**				
Obstetricians	31/52	(59.6)	1.00	1.00
Midwives/LHVs^3^	3/60	(5.0)	0.04 (0.01, 0.13)	0.02 (0.01, 0.06)
Nurses	22/131	(16.8)	0.14 (0.07, 0.28)	0.11 (0.05, 0.24)
Paediatricians	36/70	(51.4)	0.72 (0.35, 1.48)	0.49 (0.22, 1.09)
Resident doctors	72/114	(63.2)	1.16 (0.59, 2.27)	1.27 (0.63, 2.57)

**City**				
Islamabad	52/140	(37.1)	1.00	1.00
Peshawar	69/170	(40.6)	1.16 (0.73, 1.83)	3.23 (1.76, 5.91)
Rawalpindi	43/117	(36.8)	0.98 (0.59, 1.64)	0.78 (0.44, 1.37)

**Experience**				
<10 years	140/361	(38.8)	1.00	1.00
≥ 10 years	24/66	(36.4)	0.90 (0.52, 1.56)	1.36 (0.68, 2.69)

**Patients**				
<50 per day	126/341	(37.0)	1.00	1.00
≥ 50 per day	38/86	(44.2)	1.35 (0.84, 2.18)	1.41 (0.79, 2.51)

**Awareness of law**				
Unaware	105/301	(34.9)	1.00	1.00
Aware	59/126	(46.8)	1.64 (1.08, 2.51)	1.37 (0.79, 2.37)

^1^Univariate analysis, each factor considered singly

^2^Multiple logistic regression analysis, with all factors considered simultaneously

^3^LHV: lady health visitor

Resident doctors were significantly more likely than both paediatricians and obstetricians to have received free samples of infant formula (OR = 2.65, 95% CI 1.40, 5.04), and all of these highly qualified groups were significantly more likely than nurses and midwives to have been given such free samples (OR = 17.40, 95% CI 5.93, 57.42). Respondents in Rawalpindi were significantly less likely to have received free samples than those in Islamabad (OR = 0.38, 95% CI 0.16, 0.83). Receipt of free formula samples was unrelated to both length of experience and to patient workload.

Respondents who were aware of the law were significantly more likely than their colleagues who were unaware, to have accepted free samples. Of the 68 health workers who confirmed that they had received free formula samples, 28 (41.2%) were aware of the law. This difference remained statistically significant when all factors were considered together in a multivariate analysis (Table 4).

**Table 4:** Characteristics of health workers who received free samples

**Variable**	**Received/total (%)**		**Odds ratio (95% CI)**	
	
			**Unadjusted**^**1**^	**Adjusted**^**2**^
All	68/427	(15.9)		

**Occupation**				
Obstetricians	9/52	(17.3)	1.00	1.00
Midwives/LHVs^3^	0/60	(0)	0 (not estimable)	0 (not estimable)
Nurses	4/131	(3.1)	0.15 (0.04, 0.51)	0.13 (0.04,, 0.45)
Paediatricians	13/70	(18.6)	1.09 (0.43, 2.78)	0.60 (0.21, 1.70)
Resident doctors	42/114	(36.8)	2.79 (1.24, 6.28)	3.02 (1.27, 7.14)

**City**				
Islamabad	30/140	(21.4)	1.00	1.00
Peshawar	27/170	(15.9)	0.69 (0.39, 1.23)	1.37 (0.69, 2.74)
Rawalpindi	11/117	(9.4)	0.38 (0.18, 0.80)	0.26 (0.12, 0.59)

**Experience**				
<10 years	62/361	(17.2)	1.00	1.00
≥ 10 years	6/66	(9.1)	0.48 (0.20, 1.17)	0.96 (0.34, 2.70)

**Patients**				
<50 per day	50/341	(14.7)	1.00	1.00
≥ 50 per day	18/86	(20.9)	1.54 (0.85, 2.81)	1.85 (0.91, 3.77)

**Awareness of Law**				
Unaware	40/301	(13.3)	1.00	1.00
Aware	28/126	(22.2)	1.86 (1.09, 3.19)	1.99 (1.01, 3.91)

^1^Univariate analysis, each factor considered singly

^2^Multiple logistic regression analysis, with all factors considered simultaneously

^3^LHV: lady health visitor

Almost one-third of the resident doctors (27.2%, n = 31) had received sponsorship to attend training sessions or conferences compared to 8 (11.4%) paediatricians, 9 (17.3%) obstetricians, 4 (3.1%) nurses and 1 (1.7%) midwife. Resident doctors were almost 5 times more likely to have received such sponsorship compared to all other groups of health workers (OR = 4.94, 95% CI 2.72, 9.00). Highly qualified health workers were also significantly more likely to have received sponsorship than nurses and midwives (OR = 9.50, 95% CI 3.53, 27.75).

## Discussion

Although our study was too small to be representative of all urban government hospitals in Pakistan, the findings across each of the three cities were consistent in terms of awareness about the law and violations. This implies that similar findings would have also been obtained if the remaining urban cities of Pakistan were included in the study.

The study revealed that awareness about the law was very low among the health workers in the selected urban government hospitals in Pakistan. The study also revealed no adherence to the law or the Code and health workers had received gifts, free samples and sponsorship from the companies breaching the law.

These behaviours were against the provisions of articles 7.3, 7.4 & 10.2 of the national breastfeeding law [9]. Our findings coincide with a report from Pakistan that revealed companies target highly qualified health workers (paediatricians, obstetricians and resident doctors) rather than nurses or midwives/LHVs [11].

Awareness level among health workers could be improved through further training. However, the law was being violated not merely because health workers were unaware. We found that health workers were breaching the law despite being aware about it. This could be explained by non-implementation of any of the sanctions stated in the law. In most hospitals, we saw representatives from the infant formula companies distributing free samples to health staff, implying that hospital authorities had no practical rules and regulations in place to implement the law in their institutions.

It appears no rules were developed to implement the national breastfeeding legislation prior to this study. According to the minute report from the National Infant Feeding Board meeting held in June 2006, the board was still drafting the rules and regulations that would enable the practical implementation of the national breastfeeding legislation which was enacted back in 2002. Therefore, companies were continuing to flout the ban on gifts, free samples and sponsorship for health staff as it was also reported in the previous Code monitoring survey conducted in 1998 [8].

## Conclusion

Both the national breastfeeding law and the International Code of Marketing Breastmilk Substitutes were being violated in the sample hospitals in Pakistan. Implementation rules and regulations are required in the hospitals. We also recommend regular and on-going monitoring of the implementation of the law.

More studies that evaluate violations with respect to labelling formula and studies that target mothers, private and rural hospitals could further describe the implementation status.

## Competing interests

The authors declare that they have no competing interests.

## Authors' contributions

MS designed the study, participated in selecting the hospitals, conducted the interviews, analysed and interpreted data, and drafted the manuscript. AK conceived of the study, selected the hospitals, facilitated data collection and participated in the analysis and interpretation of the study. BF participated in the design of the study, critically commented on the questionnaire, the statistical analysis and interpretation of the study. PG participated in the study design, analysis and interpretation of the study and critically commented on the drafted manuscript. All authors read and approved the final manuscript.

## Supplementary Material

Additional file 1 **Questionnaire** Click here for file
