# Supplementary material for: Awareness and reported violations of the WHO International Code and Pakistan's national breastfeeding legislation; a descriptive cross-sectional survey
Source: Int Breastfeed J. 2008 Oct 17;3:24. doi: 10.1186/1746-4358-3-24 (PMC2577086; doi:10.1186/1746-4358-3-24)
Supplement: Additional file 1 — Questionnaire [file 1746-4358-3-24-S1.doc]

**Questionnaire**

My name is Mihretab M. Salasibew from Liverpool School of Tropical Medicine (LSTM) and I am conducting this study as my dissertation for the masters’ degree in community health course I am taking at LSTM.

The study aims in identifying ways in which infant formulas are being promoted in urban government hospitals in Pakistan, if at all, and in assessing health worker’s awareness with regard to efforts being made nationally and internationally to control such marketing practices. The study is being conducted as per the request submitted to LSTM from the non-governmental organization called TheNetwork for Consumers’ Protection in Pakistan.

I will be interviewing health workers like you in selected urban government hospitals in Pakistan. I appreciate if you would agree to participate in the interview. The interview would only take around ten minutes. If you need to be interviewed in a place where you would like to keep your privacy, I can do so for you. You will not be asked your name whereas the name of your hospital will be kept confidential. All your responses to the questionnaire will be stored safely and be used only for the purpose of this study.

Finally, I kindly request you to sign below if you agree to be interviewed. You may withdraw at any time during the interview if you wish to do so.

Province ………………... City ……………… Signature…………………Date……………..

**Please give your answer to each of the following questions.**

1. Gender: ………………
2. Age: ………………….
3. Current Occupation: (*Circle one*)
   1. Paediatrician
   2. Gyn/Obstetrician
   3. Resident Doctor
   4. Nurse
   5. Midwife/LHV
4. Current duty place: (*Circle one*)
   1. Antenatal care
   2. Delivery suite and postnatal care
   3. Paediatrics in-patient ward
   4. Paediatrics outpatient ward
5. How many patients or clients visit you each day? …………
6. How long have you been working in your current occupation? ………..
7. Do the infant formula companies target health workers in your hospital to promote their products? Yes: ……. No: ……….
8. If yes, what do they offer health workers? (O*ne or more answers*)
   1. Gifts (e.g. toys, pens, calendars, notepads, pencil holders etc) please specify: ………..
   2. Free samples of infant formulas
   3. Sponsorship for meetings, conference or trainings
   4. Funding for a research
   5. Cash grants
   6. Infant formula education materials
   7. Free samples of formula or any other gifts to the health workers’ families
   8. Infant formula posters, calendars or clocks to be displayed in your hospital
   9. Other, please specify …….
9. Have you ever received gifts like pens, calendars, notepads, pencil holders etc yourself? Yes…….. No…………..
10. Have you ever received free samples of infant formulas yourself? Yes ……… No ……….
11. Have you ever received any sponsorship for meetings, conferences or trainings yourself? Yes…… No ………
12. Are you aware about the existence of an International Code recommended by WHO to control marketing practices of the infant formula companies? Yes ….. No ……
13. Are you aware about the existence of national breastfeeding legislation in Pakistan? Yes ……No…….
